# Synthetic hematocrit from virtual non-contrast images for myocardial extracellular volume evaluation with photon-counting detector CT

**DOI:** 10.1007/s00330-024-10865-7

**Published:** 2024-06-27

**Authors:** Victor Mergen, Nicolas Ehrbar, Lukas J. Moser, Johannes C. Harmes, Robert Manka, Hatem Alkadhi, Matthias Eberhard

**Affiliations:** 1 Diagnostic and Interventional Radiology, University Hospital Zurich, University of Zurich, Zurich, Switzerland; 2 Department of Cardiology, University Heart Center, University Hospital Zurich, University of Zurich, Zurich, Switzerland; 3 Radiology, Spitäler fmi AG, Spital Interlaken, Unterseen, Switzerland

**Keywords:** Cardiac imaging, Photon-counting detector CT, Spectral imaging, Extracellular volume

## Abstract

**Objectives:**

To assess the accuracy of a synthetic hematocrit derived from virtual non-contrast (VNC) and virtual non-iodine images (VNI) for myocardial extracellular volume (ECV) computation with photon-counting detector computed tomography (PCD-CT).

**Materials and methods:**

Consecutive patients undergoing PCD-CT including a coronary CT angiography (CCTA) and a late enhancement (LE) scan and having a blood hematocrit were retrospectively included. In the first 75 patients (*derivation cohort*), CCTA and LE scans were reconstructed as VNI at 60, 70, and 80 keV and as VNC with quantum iterative reconstruction (QIR) strengths 2, 3, and 4. Blood pool attenuation (BP_mean_) was correlated to blood hematocrit. In the next 50 patients (*validation cohort*), synthetic hematocrit was calculated using BP_mean_. Myocardial ECV was computed using the synthetic hematocrit and compared with the ECV using the blood hematocrit as a reference.

**Results:**

In the derivation cohort (49 men, mean age 79 ± 8 years), a correlation between BP_mean_ and blood hematocrit ranged from poor for VNI of CCTA at 80 keV, QIR2 (*R*^2^ = 0.12) to moderate for VNI of LE at 60 keV, QIR4; 70 keV, QIR3 and 4; and VNC of LE, QIR3 and 4 (all, *R*^2^ = 0.58). In the validation cohort (29 men, age 75 ± 14 years), synthetic hematocrit was calculated from VNC of the LE scan, QIR3. Median ECV was 26.9% (interquartile range (IQR), 25.5%, 28.8%) using the blood hematocrit and 26.8% (IQR, 25.4%, 29.7%) using synthetic hematocrit (VNC, QIR3; mean difference, −0.2%; limits of agreement, −2.4%, 2.0%; *p* = 0.33).

**Conclusion:**

Synthetic hematocrit calculated from VNC images enables an accurate computation of myocardial ECV with PCD-CT.

**Clinical relevance statement:**

Virtual non-contrast images from cardiac late enhancement scans with photon-counting detector CT allow the calculation of a synthetic hematocrit, which enables accurate computation of myocardial extracellular volume.

**Key Points:**

- *Blood hematocrit is mandatory for conventional myocardial extracellular volume computation.*
- *Synthetic hematocrit can be calculated from virtual non-iodine and non-contrast photon-counting detector CT images.*
- *Synthetic hematocrit from virtual non-contrast images enables computation of the myocardial extracellular volume.*

## Introduction

The myocardial extracellular volume (ECV) quantifies the extent of myocardial interstitial fibrosis and is a promising biomarker reflecting left ventricular function [1–3]. For example, in patients with severe aortic valve stenosis, the progression of stenosis leads to chronic pressure overload of the left myocardium eventually leading to remodeling of the left ventricle with the development of fibrosis [1, 4]. In addition, cardiac amyloidosis occurs with a high prevalence in patients with severe aortic stenosis and can be diagnosed by globally increased ECV [5]. Thus, recent studies proposed comprehensive computed tomography (CT) protocols for the diagnostic work-up of patients planned to undergo transcatheter aortic valve replacement (TAVR) including late enhancement (LE) scans for the assessment of the myocardial ECV, since elevated myocardial ECV has been identified as an independent risk factor of mortality in patients after TAVR [4, 6–10].

Photon-counting detector computed tomography (PCD-CT) has the potential to refine the cardiac assessment in patients prior to TAVR owing to the possibility of acquiring dual-source cardiac scans with ultra-high resolution or with spectral information at a maintained high temporal resolution [11–17]. Ultra-high-resolution coronary CT angiography (CCTA) reduces calcium-induced blooming artifacts and provides high diagnostic accuracy for the detection of significant stenosis even in patients with severe coronary artery calcifications [13, 18, 19]. Cardiac LE scans with spectral information allow the computation of myocardial ECV based on iodine images only, obviating the need for unenhanced cardiac scans, which are mandatory when using a conventional, attenuation-based method [20, 21]. ECV quantification using iodine maps of LE scans with PCD-CT showed a strong correlation to cardiac magnetic resonance imaging (MRI), which is the reference standard for tissue characterization [20].

The timeliness of the blood hematocrit is critical, as it constitutes an integral part of the myocardial ECV calculation [3, 20]. Unfortunately, blood hematocrit is not always available at the time of the scan. However, attenuation of the blood pool determined on virtual non-contrast (VNC) images from PCD-CT indicated to allow accurate quantification of hemoglobin levels [22, 23].

The aim of the study was to assess the accuracy of synthetic hematocrit from VNC and virtual non-iodine (VNI) images for myocardial ECV computation with PCD-CT.

## Materials and methods

### Patients

**Fig. 1:**
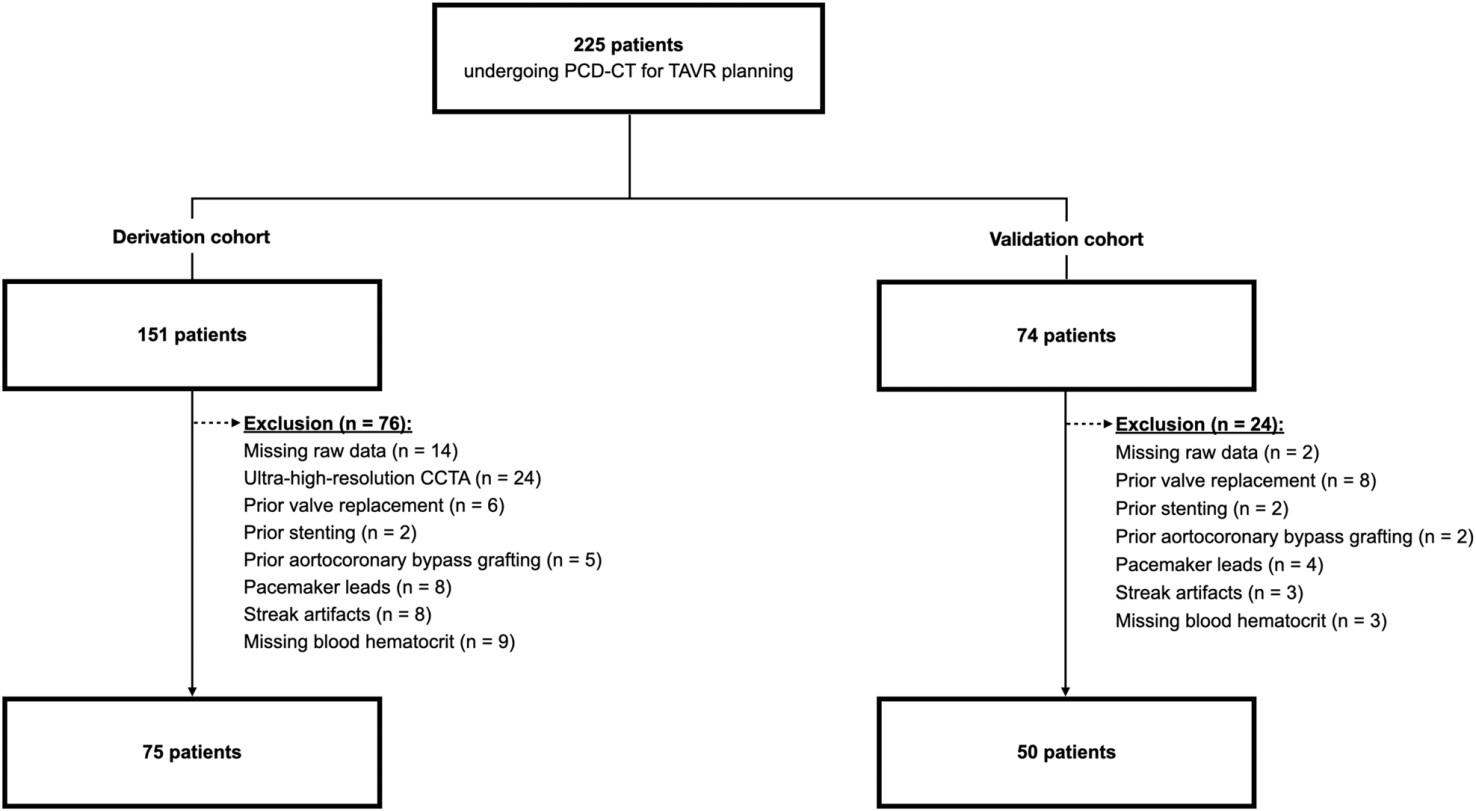
Flowchart detailing patient inclusion. CCTA, coronary CT angiography; PCD-CT, photon-counting detector CT; TAVR, transcatheter aortic valve replacement

In this retrospective, single-center study, consecutive patients with severe aortic stenosis undergoing a PCD-CT, which comprised a CCTA and a cardiac LE scan between May 2022 and November 2023 were screened. Scans were acquired as part of the diagnostic work-up for TAVR planning. Exclusion criteria were missing raw data, prior valve replacement, coronary stenting, aortocoronary bypass grafting, or pacemaker leads as foreign material may impede measurements, severe streak artifacts from gross calcifications, and missing blood hematocrit. Studies with an ultra-high-resolution CCTA were also excluded. The first 75 patients were used as a derivation cohort. The following 50 patients served as a validation cohort (Fig. 1).

All patients provided written informed consent to allow the inclusion of their anonymized data in retrospective analyses. Institutional review board and local ethics committee approval were obtained.

### CT data acquisition and image reconstruction

Scans were performed on a first-generation dual-source PCD-CT system (NAEOTOM Alpha; Siemens Healthineers AG) equipped with two cadmium telluride detectors. The scan protocol started with an electrocardiography (ECG)-gated non-contrast cardiac scan, followed by an ECG-gated CCTA, a whole-body aortography, and ending with an ECG-gated cardiac LE scan. CCTA was initiated after the injection of a weight-based volume of iodinated contrast medium (60–80 mL, iopromide, Ultravist 370 mgI/mL; Bayer Healthcare) accompanied by a saline chaser (20 mL, NaCl 0.9%) into an antecubital vein applying a weight-based flow rate (3.3–4.4 mL/s). Cardiac LE scans were acquired five minutes after contrast media injection.

The ECG-gated sequential multi-energy QuantumPlus mode with a detector collimation of 144 × 0.4 mm was applied for CCTA and LE scans. CCTA and LE scans were acquired with a tube potential of 140 kV and 120 kV, respectively, both using automated tube current modulation (CAREDose4D, Siemens) with an image quality level of 64 and 80, respectively. The ECG-pulsing window was fixed from 30% to 60% of the R-R interval for the CCTA and to an absolute interval of 280 milliseconds from the R wave for the LE scan. The gantry rotation time was 0.25 s. No beta-blockers were administered.

### Derivation cohort—image reconstruction and linear regression analysis

**Fig. 2:**
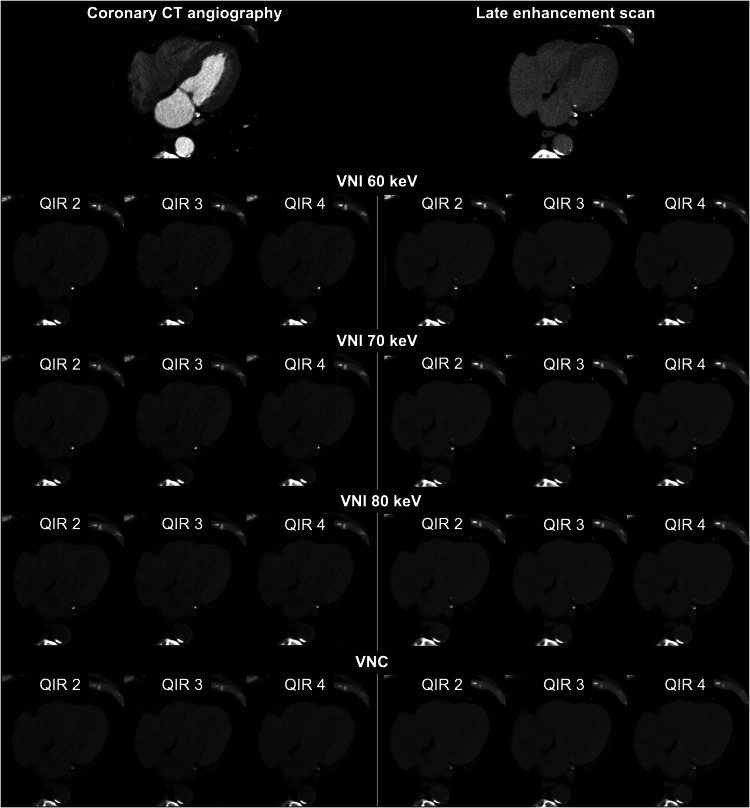
Representative CT images obtained in an 85-year-old man with severe aortic stenosis examined for transcatheter aortic valve replacement planning. Axial CT images acquired with dual-source photon-counting detector CT show the virtual non-iodine (VNI) images at 60, 70, and 80 keV and the virtual non-contrast (VNC) images reconstructed from coronary CT angiography and cardiac late enhancement scan with quantum iterative reconstruction (QIR) strengths 2, 3, and 4

CCTA and cardiac LE scans were reconstructed as calcium-preserving VNI images at 60, 70, and 80 keV [14, 24] as well as VNC images [22, 25] applying quantum iterative reconstruction (QIR) strengths 2, 3, and 4 [26], resulting in a total of 24 reconstructions for every patient (Fig. 2). VNI and VNC images were reconstructed with a slice thickness of 3 mm and increment of 1.5 mm using the quantitative Qr36 kernel. All images were reconstructed with a field of view of 200 × 200 mm^2^ and using a matrix size of 512 × 512 pixels.

One reader (N.E.), who was blinded to clinical information including the blood hematocrit, measured the CT attenuation of the blood pool by placing circular regions of interest (ROIs) in the left ventricle, and in the ascending and descending aorta at the level of the right pulmonary artery. The ROIs were first positioned in CCTA with the largest possible diameter while carefully avoiding adjacent structures and were then copied to VNI and VNC images from CCTA and LE scans. All image sets were linked to show identical anatomical structures. Mean blood pool attenuation (BP_mean_) was calculated by averaging the attenuation values of all three regions. BP_mean_ of every reconstruction was compared with the blood hematocrit with linear regression analyses and Pearson correlation.

### Validation cohort—image reconstruction, synthetic hematocrit calculation, and extracellular volume computation

Cardiac LE scans were reconstructed as VNC images applying QIR 3 according to the results of the derivation cohort and supported by previous studies [22, 23] to calculate the synthetic hematocrit. Moreover, specific images were reconstructed from CCTA and cardiac LE scans to allow for the computation of the myocardial ECV: virtual monoenergetic images (VMI) at 65 keV from CCTA and LE scans, and iodine images from LE scans. The ECV-specific images were reconstructed with a slice thickness of 1.5 mm and increment of 1 mm, using the quantitative Qr40 kernel and applying QIR 3 as detailed in previous studies [20, 21]. All images were reconstructed with a field of view of 200 × 200 mm^2^ and using a matrix size of 512 × 512 pixels. Supplemental Fig. 1 delineates the various reconstructed images and their corresponding tasks.

The same reader (N.E.) measured BP_mean_ in a single image set. The synthetic hematocrit was calculated using BP_mean_ obtained as described above, employing the corresponding linear regression formula determined in the derivation cohort. A second reader (V.M.) computed the myocardial ECV with a semi-automatic software (Cardiac Functional Analysis Frontier Version 2.1.0, Syngo.via, Siemens Healthineers AG) applying the iodine image-derived iodine concentration in the myocardium and the blood pool of the LE scan [3]:

$${{{{{{\mathrm{Myocardial}}}}}}\; {{{{{\mathrm{ECV}}}}}}}=\left(1-{{{{{{\mathrm{hematocrit}}}}}}}\right)* \frac{[{{{{{{{\mathrm{Iodine}}}}}}}}_{{{{{{{\mathrm{myocardium}}}}}}}}]}{[{{{{{{{\mathrm{Iodine}}}}}}}}_{{{{{{{\mathrm{blood}}}}}}\; {{{{{\mathrm{pool}}}}}}}}]}$$

For myocardial ECV calculation, either the blood hematocrit (determined via blood drawing) or a synthetic hematocrit (derived from ROI measurements directly on the images as described above) served as input values. Mean midmyocardial ECV, which included the myocardium from the inner 25% to the outer 25%, was noted.

### Statistical analysis

Analyses were performed using R statistical software (R, version 4.3.2; R Foundation, https://www.R-project.org/). Variables were tested for normal distribution with the Shapiro-Wilk test. Continuous variables were summarized as means and standard deviations or medians and interquartile ranges when normally or nonnormally distributed. Categorical variables were presented as counts and percentages. In the derivation cohort, BP_mean_ was correlated to the blood hematocrit with linear regression analyses and Pearson correlations [27]. In the validation cohort, myocardial ECV using the blood hematocrit and the synthetic hematocrit were compared with Bland-Altman analyses and Wilcoxon signed-rank tests. Patient characteristics and blood hematocrit between the derivation and validation cohorts were compared using *t*-tests and the Wilcoxon signed-rank tests, as appropriate. A two-tailed *p*-value less than 0.05 was considered to indicate statistical significance.

## Results

### Patient sample

Of 225 consecutive patients scanned for TAVR planning, 76 patients were excluded in the derivation cohort and 24 patients were excluded in the validation cohort (Fig. 1). Finally, the derivation cohort consisted of 26 women and 49 men (mean age 79 ± 8 years, mean body mass index (BMI) 26.4 ± 5.1 kg/m^2^) and the validation cohort of 21 women and 29 men (mean age 78 ± 8 years, mean BMI 25.4 ± 4.9 kg/m^2^) (Table 1). Patient characteristics were similar in the derivation and the validation cohorts (all, *p* > 0.05). Mean blood hematocrit was 0.381 ± 0.058 L/L in the derivation cohort and 0.387 ± 0.043 L/L in the validation cohort (*p* = 0.52). Both the blood test and the scan were performed on the same day in 60/75 patients (80%) and within one day in 67/75 patients (89%) in the derivation cohort. In the validation cohort, both the blood test and the scan were performed on the same day in 35/50 patients (70%) and within one day in 43/50 patients (86%). Radiation dose parameters are presented in Table 1.

**Table 1:** Patient demographics

	Derivation cohort (*n* = 75)	Validation cohort (*n* = 50)	*p*-value
*Patient characteristics*			
Sex			0.41
Female	26/75 (35)	21/50 (42)	
Male	49/75 (65)	29/50 (58)	
Age (years)^a^	79 ± 8 (range, 58–94)	78 ± 8 (range, 60–94)	0.18
Body mass index (kg/m^2^)^a^	26.4 ± 5.1 (range, 16.8–40.6)	25.8 ± 4.8 (range, 16.6–43.0)	0.36
Heart rate during scan acquisition (bpm)	76 ± 20 (range, 51–171)	74 ± 16 (range, 48–119)	0.60
*Medical history*			
Arterial hypertension	58/75 (77)	30/50 (60)	
Dyslipidemia	42/75 (56)	24/50 (48)	
Diabetes	37/75 (49)	11/50 (22)	
Smoking	31/75 (41)	13/50 (26)	
Chronic kidney disease	27/75 (36)	14/50 (28)	
*Blood test*			
Hematocrit (L/L)	0.381 ± 0.058	0.387 ± 0.043	0.52
*Radiation dose parameters*			
Coronary CT angiography			
CTDI_vol_ (mGy^)b^	14.0 (11.1, 20.4)		
DLP (mGy·cm)^b^	198 (142, 276)		
Late enhancement scan			
CTDI_vol_ (mGy)^b^	7.3 (5.9, 9.0)	6.8 (5.9, 8.2)	0.23
DLP (mGy·cm)^b^	105 (85, 120)	94 (80, 116)	0.15

Unless otherwise stated, data are numbers of participants, with percentages in parentheses *bpm* beats per minute, *CTDI*_*vol*_ volume CT dose index, *DLP* dose length product

^a^Data are means ± SDs

^b^Data are medians and interquartile ranges within parentheses

### Regression analyses and Pearson correlations

In the derivation cohort, correlation was moderate between blood hematocrit and BP_mean_ for all VNI and VNC images from LE scans regardless of the monoenergetic level and the QIR strength (Pearson *R*^2^, 0.56–0.58, all, *p* < 0.001, Table 2). Highest Pearson *R*^2^ of 0.58 was found for VNI images at 60 keV with QIR 4, VNI images at 70 keV with QIR 3 and 4, and all VNC images regardless of the QIR strength. Blood hematocrit and BP_mean_ showed a very weak correlation using VNI and VNC images from CCTA regardless of the monoenergetic level and the QIR strength (Pearson *R*^2^, 0.12–0.16, all, *p* < 0.001).

**Table 2:** Pearson correlations of the validation cohort

Image reconstruction	keV	QIR	Coronary CT angiography		Late enhancement scan	
			Pearson *R*^*2*^	*p*-value	Pearson *R*^*2*^	*p*-value
VNI	60	2	0.16	< 0.001	0.57	< 0.001
		3	0.15	< 0.001	0.57	< 0.001
		4	0.16	< 0.001	0.58	< 0.001
	70	2	0.16	< 0.001	0.57	< 0.001
		3	0.16	< 0.001	0.58	< 0.001
		4	0.16	< 0.001	0.58	< 0.001
	80	2	0.12	0.003	0.57	< 0.001
		3	0.16	< 0.001	0.57	< 0.001
		4	0.16	< 0.001	0.56	< 0.001
VNC		2	0.16	< 0.001	0.58	< 0.001
		3	0.16	< 0.001	0.58	< 0.001
		4	0.15	< 0.001	0.58	< 0.001

*VNC* virtual non-contrast, *VNI* virtual non-iodine, *QIR* quantum iterative reconstruction

**Fig. 3:**
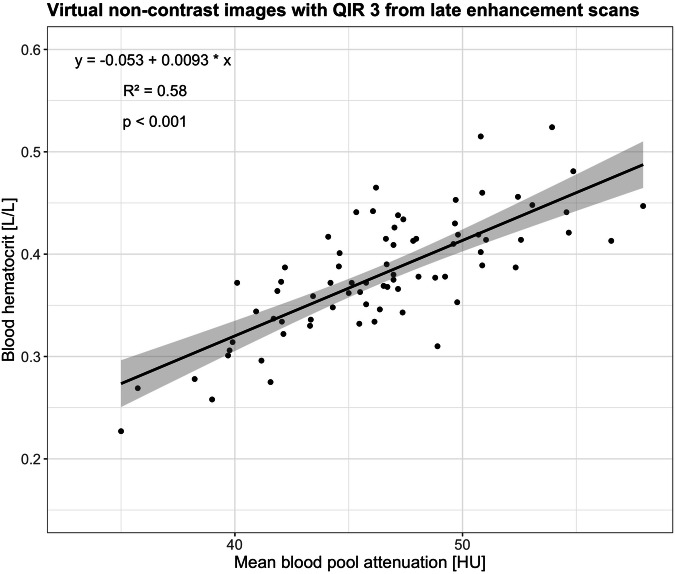
Linear regression and Pearson correlation of the blood hematocrit and the mean blood pool attenuation using virtual non-contrast images (VNC) with quantum iterative reconstruction (QIR) strength 3, which showed the highest degree of correlation to the blood hematocrit. The solid line corresponds to the regression line, and the shaded areas indicate the 95% confidence intervals for the regression line

Linear regression analysis for VNC images from LE scans with QIR 3 is presented in Fig. 3.

### Extracellular volume computation

In the validation cohort, median myocardial ECV was 26.9% (interquartile range (IQR), 25.5%, 28.8%) using the blood hematocrit and 26.8% (IQR, 25.4%, 29.7%) using the synthetic hematocrit derived from VNC images of LE scans with QIR 3.

**Fig. 4:**
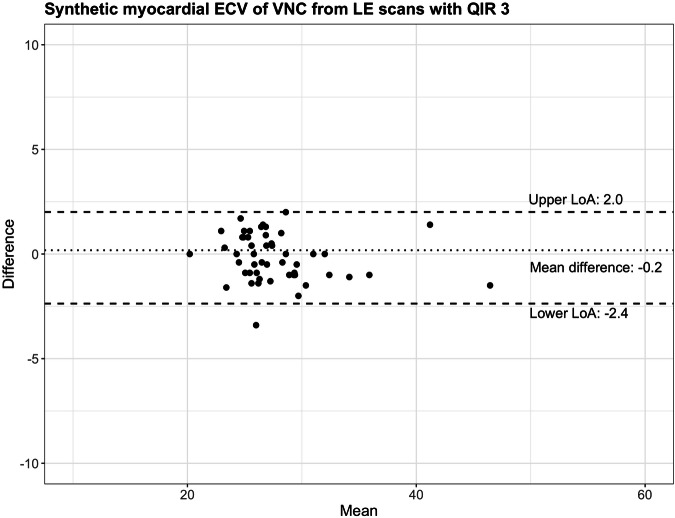
Bland-Altman plot comparing myocardial extracellular volumes (ECV) using blood hematocrit and synthetic hematocrit from virtual non-contrast images (VNC) of cardiac late enhancement (LE) scans with quantum iterative reconstruction (QIR) strength 3. LoA, limit of agreement

**Fig. 5:**
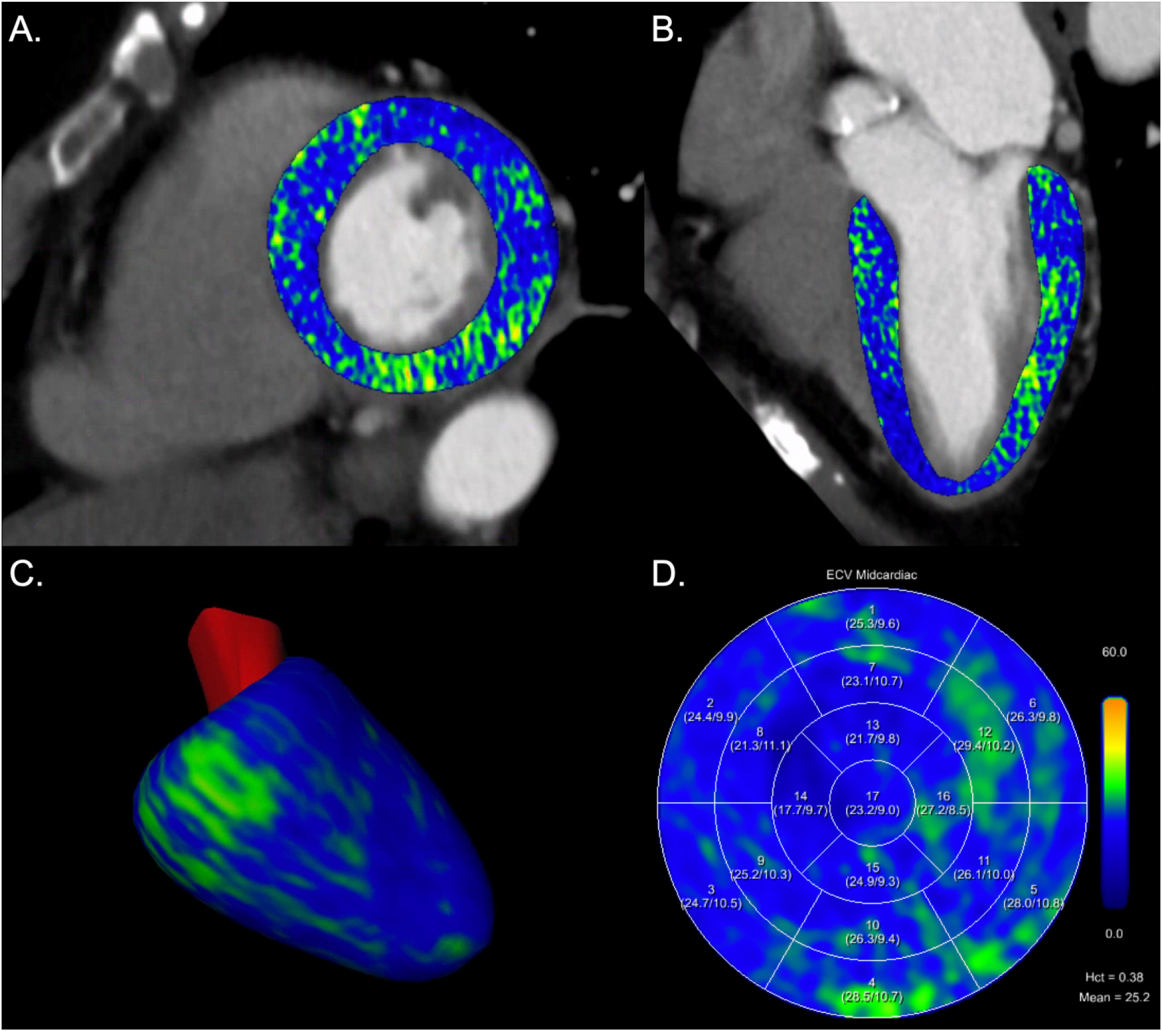
Myocardial extracellular volume (ECV) computation in a 72-year-old female patient with severe aortic stenosis. Images are displayed as short axis (**A**) and 2-chamber views (**B**) of extracellular maps calculated from the late enhancement scan superimposed on the coronary CT angiography images, 3D model of the left ventricle (**C**), and ECV polar map according to the 17-segment model of the American Heart Association (**D**). Blood hematocrit was 0.383 L/L resulting in a mean midmyocardial ECV of 25.2%; synthetic hematocrit was 0.402 L/L resulting in a mean midmyocardial ECV of 24.4%. Hct, hematocrit

**Fig. 6:**
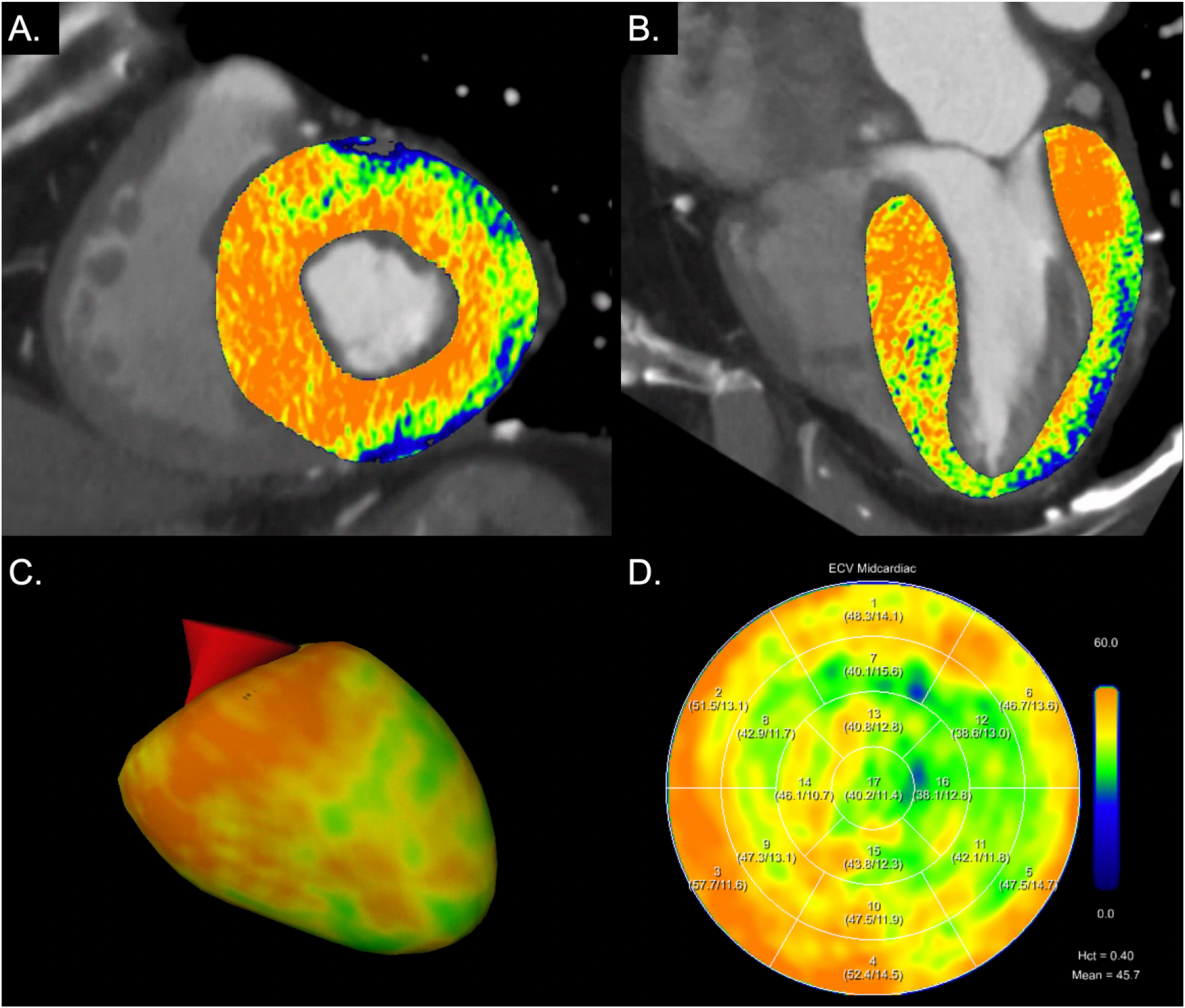
Myocardial extracellular volume (ECV) computation in an 84-year-old male patient with severe aortic stenosis. Images are displayed as short axis (**A**) and 2-chamber views (**B**) of extracellular maps calculated from the late enhancement scan superimposed on coronary CT angiography images, 3D model of the left ventricle (**C**), and ECV polar map according to the 17-segment model of the American Heart Association (**D**). Blood hematocrit was 0.404 L/L resulting in a mean midmyocardial ECV of 45.7%; synthetic hematocrit was 0.382 L/L resulting in a mean midmyocardial ECV of 47.2%. Myocardial ECV was elevated, while ECV values at the apex were lower (apical sparing), indicating cardiac amyloidosis. Hct, hematocrit

Myocardial ECVs were similar between methods (*p* = 0.33) with a mean difference of −0.2% (lower limit of agreement −2.4%, upper limit of agreement 2.0%, Fig. 4). Figures 5 and 6 provide representative examples of myocardial ECV calculations.

## Discussion

Myocardial ECV provides incremental prognostic information in patients with severe aortic valve stenosis undergoing TAVR [4, 6]. Cardiac scans from PCD-CT allow for the assessment of myocardial ECV based on spectral LE scans, but its computation requires a recent blood hematocrit. First, in a derivation cohort, VNI and VNC images from CCTA or cardiac LE scans with the highest correlation to the blood hematocrit were determined. Second, in an independent validation cohort, myocardial ECV was computed using the synthetic hematocrit derived from the previously determined reconstruction and compared with the ECV using the blood hematocrit as a reference. The myocardial ECV computed using the synthetic hematocrit derived from VNC images of cardiac LE scans with QIR 3 showed no significant difference compared with the ECV using the blood hematocrit (*p* = 0.33).

Decker et al evaluated the feasibility and accuracy of diagnosing anemia using VNC images reconstructed from contrast-enhanced thoracoabdominal CT scans acquired in the portal venous phase with a PCD-CT system [22, 23]. Blood pool attenuation had a strong correlation to blood hematocrit (*R*^2^ = 0.77, [22] and *R*^2^ = 0.76 [23]), which enabled the detection of anemia. In the study presented here, a more comprehensive evaluation was performed additionally including VNI images at three monoenergetic levels with three QIR strengths as well as VNC images with three QIR strengths from dual-source spectral CCTA and cardiac LE scans. Both the VNI (PureCalcium, Siemens) and VNC (VNC, Siemens) algorithms have been developed to virtually subtract iodine-based contrast media from spectral contrast-enhanced scans [24, 25, 28, 29]. The VNI algorithm operates by decomposing images into the base materials iodine and calcium, with the specific aim of preserving vascular and cardiac calcifications as well as stent structures for vascular imaging [24, 28, 29]. This algorithm initially identifies calcified structures and subsequently proceeds with a decomposition into the materials iodine and calcium within these regions. The VNC algorithm operates by decomposing images into the base materials water and iodine, and is tailored to preserve the CT attenuation in parenchymal structures [25]. In the derivation cohort, the highest correlation between blood hematocrit and BP_mean_ was observed using VNI images at 60 keV with QIR 4, at 70 keV with QIR 3 and 4, and all VNC images regardless of the QIR strength from spectral cardiac LE scans (all, *R*^2^ = 0.58). Compared with these previous studies [22, 23], various dissimilarities may have influenced the correlation coefficients. Decker et al quantified blood pool attenuation across five different regions, namely the left atrium, left ventricle, pulmonary trunk, ascending aorta, and descending aorta. In contrast, the present study streamlined measurements to three regions, specifically the left ventricle, ascending aorta, and descending aorta. Additionally, Decker et al reconstructed the VNC images from contrast-enhanced thoracoabdominal scans acquired in the portal venous phase using the single-source spectral mode, whereas this study reconstructed VNI and VNC images from CCTA and cardiac LE scans using the dual-source spectral mode.

The calculation of myocardial ECV using a synthetic hematocrit derived from blood pool measurements was first introduced and validated for cardiac MRI [30]. Treibel et al extended this concept to cardiac CT showing that the calculation of myocardial ECV using a synthetic hematocrit derived from blood pool attenuation on unenhanced chest images was feasible [31]. In their study, the mean difference between myocardial ECV using blood and synthetic hematocrit was 2.4% with a standard deviation of 5.7% [31]. Kim et al proposed the calculation of myocardial ECV with a synthetic hematocrit derived from virtual unenhanced images using dual-energy cardiac LE scans acquired with a conventional energy-integrating detector CT and found similar results compared to myocardial ECV from MRI [32]. The formula for the calculation of a synthetic hematocrit presented by Kim et al highly differs from the formula issued from the derivation cohort of our study: synthetic hematocrit = 0.85 × (VUE attenuation of blood) − 5.40 in the study by Kim et al [32] and synthetic hematocrit = 0.0093 × blood pool attenuation on VNC images − 0.053 in our study. Given the differences in the virtual iodine removal algorithms used for dual-energy scans acquired with conventional scanners and spectral scans acquired with PCD-CT, it is inappropriate to directly compare the formulae from the two methods. In our study, the myocardial ECV computation revealed a mean difference of −0.2% between values obtained using blood and synthetic hematocrit with narrow limits of agreement (lower limit, −2.4%; upper limit, 2.0%). These findings underscore the capability of spectral cardiac scans employing PCD-CT to accurately compute myocardial ECV without the necessity of drawing the blood hematocrit in each patient. Confirmation of these observations by future studies is certainly imperative.

The following study limitations merit consideration. First, in this retrospective, single-center study, both the derivation and the validation cohort only included a limited number of patients. Second, same-day blood hematocrit was not available in all patients. Third, VNI and VNC images were reconstructed using a specific set of parameters, chosen based on the findings of previous studies and personal expertise [24, 33]. Fourth, the impact of dual-source ECG-gated helical or high-pitch scan mode on myocardial ECV with synthetic hematocrit was not evaluated as both CCTA and LE scans were acquired in the sequential mode. Fifth, synthetic hematocrit was computed using only one VNC reconstruction from the LE scan. Finally, CT-derived myocardial ECV was not compared to the clinical reference standard cardiac MRI. However, a previous study showed a high correlation between myocardial ECV using iodine maps of LE scans with PCD-CT and cardiac MRI [20].

In conclusion, our study in patients with severe aortic stenosis indicates that synthetic hematocrit derived from virtual non-contrast images of spectral LE CT scans enables an accurate calculation of myocardial ECV, potentially obviating the need for a blood hematocrit test in each patient.

## Supplementary information

Electronic Supplementary Material

## Abbreviations

CT: Computed tomography
CCTA: Coronary CT angiography
ECV: Extracellular volume
LE: Late enhancement
MRI: Magnetic resonance imaging
PCD-CT: Photon-counting detector CT
QIR: Quantum iterative reconstruction
VNC: Virtual non-contrast
VNI: Virtual non-iodine images
