# Supplementary material for: Synthetic hematocrit from virtual non-contrast images for myocardial extracellular volume evaluation with photon-counting detector CT
Source: Eur Radiol. 2024 Jun 27;34(12):7845–55. doi: 10.1007/s00330-024-10865-7 (PMC11557661; doi:10.1007/s00330-024-10865-7)
Supplement: Supplementary file 1 — Electronic Supplementary Material [file 330_2024_10865_MOESM1_ESM.pdf]

Synthetic hematocrit from virtual non-contrast images for myocardial extracellular volume evaluation with photon-counting detector CT

Electronic Supplementary Material

Eur Radiol (2024) Mergen V, Ehrbar N, Moser LJ et al.

Supplemental Figure 1: Flowchart delineating the various reconstructed images and their corresponding tasks in the derivation and the validation cohort.

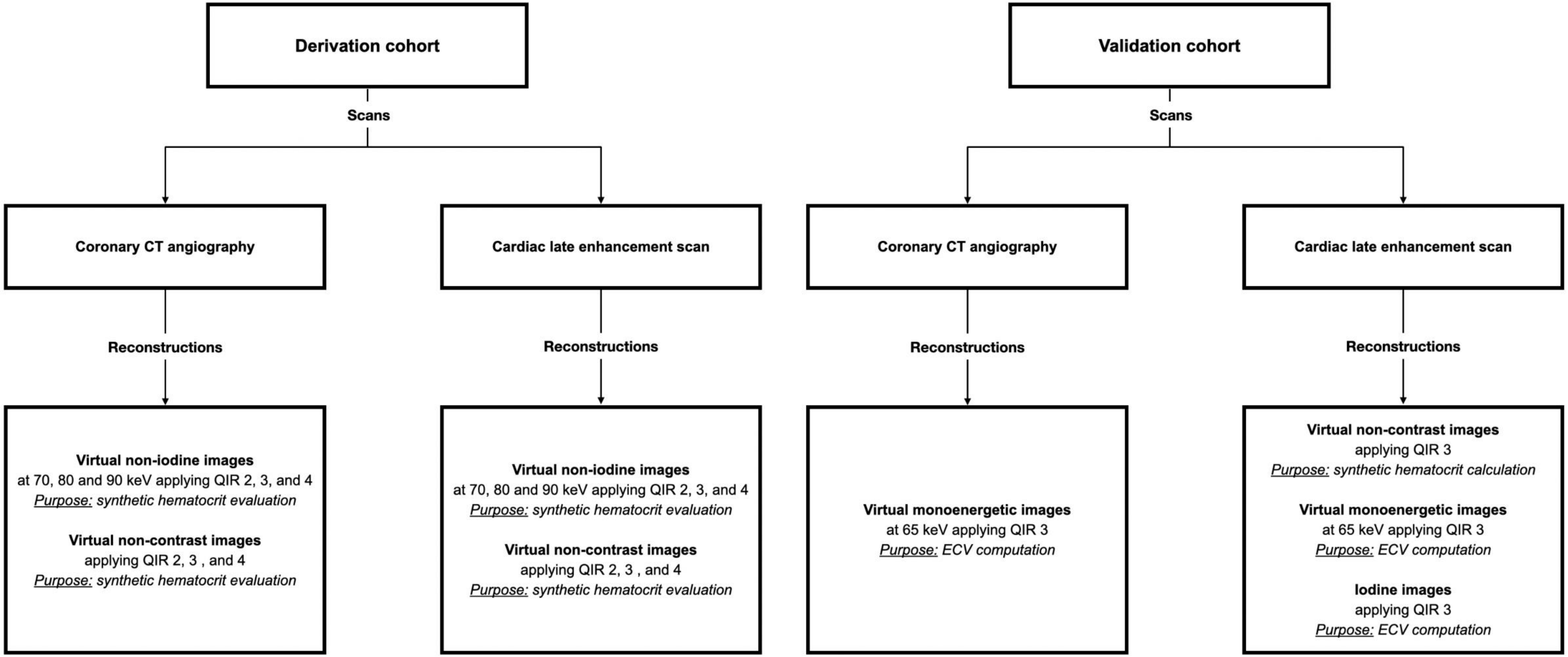

ECV = extracellular volume, QIR = quantum iterative reconstruction
